# IncA/C Conjugative Plasmids Mobilize a New Family of Multidrug Resistance Islands in Clinical *Vibrio cholerae* Non-O1/Non-O139 Isolates from Haiti

**DOI:** 10.1128/mBio.00509-16

**Published:** 2016-07-19

**Authors:** Nicolas Carraro, Nicolas Rivard, Daniela Ceccarelli, Rita R. Colwell, Vincent Burrus

**Affiliations:** a Laboratory of Bacterial Molecular Genetics, Département de Biologie, Faculté des Sciences, Université de Sherbrooke, Sherbrooke, Quebec, Canada; b Central Veterinary Institute of Wageningen UR, Lelystad, The Netherlands; c Maryland Pathogen Research Institute, University of Maryland, College Park, Maryland, USA; d Johns Hopkins Bloomberg School of Public Health, Johns Hopkins University, Baltimore, Maryland, USA; e Center for Bioinformatics and Computational Biology, University of Maryland Institute for Advanced Computer Studies (UMIACS), University of Maryland, College Park, Maryland, USA

## Abstract

Mobile genetic elements play a pivotal role in the adaptation of bacterial populations, allowing them to rapidly cope with hostile conditions, including the presence of antimicrobial compounds. IncA/C conjugative plasmids (ACPs) are efficient vehicles for dissemination of multidrug resistance genes in a broad range of pathogenic species of *Enterobacteriaceae*. ACPs have sporadically been reported in *Vibrio cholerae*, the infectious agent of the diarrheal disease cholera. The regulatory network that controls ACP mobility ultimately depends on the transcriptional activation of multiple ACP-borne operons by the master activator AcaCD. Beyond ACP conjugation, AcaCD has also recently been shown to activate the expression of genes located in the *Salmonella* genomic island 1 (SGI1). Here, we describe MGI*Vch*Hai6, a novel and unrelated mobilizable genomic island (MGI) integrated into the 3′ end of *trmE* in chromosome I of *V. cholerae* HC-36A1, a non-O1/non-O139 multidrug-resistant clinical isolate recovered from Haiti in 2010. MGI*Vch*Hai6 contains a mercury resistance transposon and an integron In104-like multidrug resistance element similar to the one of SGI1. We show that MGI*Vch*Hai6 excises from the chromosome in an AcaCD-dependent manner and is mobilized by ACPs. Acquisition of MGI*Vch*Hai6 confers resistance to β-lactams, sulfamethoxazole, tetracycline, chloramphenicol, trimethoprim, and streptomycin/spectinomycin. *In silico* analyses revealed that MGI*Vch*Hai6-like elements are carried by several environmental and clinical *V. cholerae* strains recovered from the Indian subcontinent, as well as from North and South America, including all non-O1/non-O139 clinical isolates from Haiti.

## IMPORTANCE

*Vibrio cholerae*, the causative agent of cholera, remains a global public health threat. Seventh-pandemic *V. cholerae* acquired multidrug resistance genes primarily through circulation of SXT/R391 integrative and conjugative elements. IncA/C conjugative plasmids have sporadically been reported to mediate antimicrobial resistance in environmental and clinical *V. cholerae* isolates. Our results showed that while IncA/C plasmids are rare in *V. cholerae* populations, they play an important yet insidious role by specifically propagating a new family of genomic islands conferring resistance to multiple antibiotics. These results suggest that nonepidemic *V. cholerae* non-O1/non-O139 strains bearing these genomic islands constitute a reservoir of transmissible resistance genes that can be propagated by IncA/C plasmids to *V. cholerae* populations in epidemic geographical areas as well to pathogenic species of *Enterobacteriaceae*. We recommend future epidemiological surveys take into account the circulation of these genomic islands.

## INTRODUCTION

The diarrheal disease cholera remains a serious public health threat worldwide (1). The O1 and O139 toxigenic strains of *Vibrio cholerae*, the etiologic agent of cholera, produce a toxin that causes profuse diarrhea, vomiting, and subsequent severe dehydration (2). While proper hydration is usually sufficient to treat cholera patients, antibiotic therapy is necessary to treat severe cases and to reduce the release of this infectious agent into the environment (1). Toxigenic *V. cholerae* is very efficient at rapidly infecting and spreading among and between human populations (1, 3). Stepwise evolution of pathogenic lineages of *V. cholerae* has involved acquisition of adaptive traits, such as antibiotic resistance genes and virulence factors encoded by diverse mobile genetic elements, including those coding for cholera toxin and carried on the CTXφ phage, pathogenicity islands, and integrative and conjugative elements (ICEs) (2, 4–7).

ICEs of the SXT/R391 family have been recognized as major drivers of the dissemination of antibiotic resistance genes among several species of *Enterobacteriaceae* and *Vibrionaceae*, including environmental and clinical *V. cholerae* isolates (8–10). ICE*Vch*Ind5, an ICE conferring resistance to sulfamethoxazole-trimethoprim (co-trimoxazole), streptomycin, and chloramphenicol has been shown to be the most prevalent variant of SXT/R391 ICE in the seventh-pandemic multidrug-resistant *V. cholerae* lineage (5–7, 11). While the role of SXT/R391 ICEs has been extensively studied over the last two decades, recent studies have also highlighted the sporadic involvement of conjugative plasmids of the IncA/C group (ACPs) in genome plasticity and multidrug resistance (MDR) acquisition in *V. cholerae* (12–18). ACPs are large plasmids (>110 kb) that transfer efficiently by conjugation to and maintenance in a broad range of *Gammaproteobacteria* (19, 20). ACPs are a threat to human and animal health due to their worldwide prevalence in clinical isolates of bacterial enteric pathogens and their carriage of a large variety of antibiotic resistance genes (18, 19, 21–24). Several reports have associated ACPs with resistance to penicillins, cephalosporins, and carbapenems, conferred by allelic variants of the *bla*_NDM_ gene (25–28). These plasmids are frequently detected in food products, food-producing animals, and environmental samples that likely constitute a large reservoir for their subsequent dissemination to human pathogens (19).

**FIG 1:**
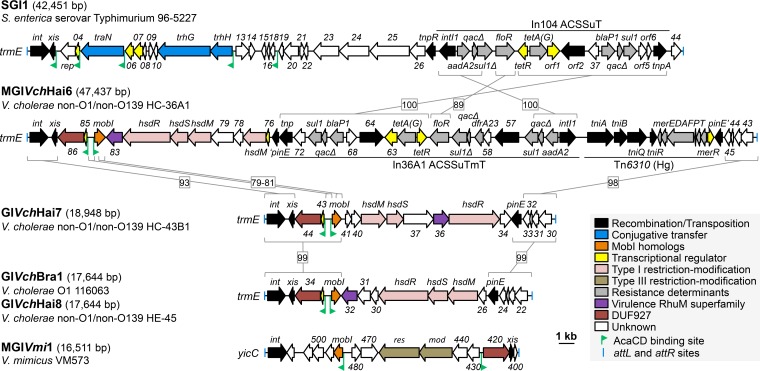
AcaCD-activated GIs. Schematic representation of the genetic map of GIs bearing predicted AcaCD binding sites. GIs are drawn to scale. The left and right junctions (*attL* and *attR*) within the host chromosome are indicated by blue bars at the extremities. ORFs with similar function are color coded as indicated in the figure. Green chevrons indicate the position and orientation of predicted AcaCD-binding sites. Homologous regions are bracketed and linked by a gray line with the corresponding percentage of nucleotide identity. Gene numbers correspond to the last digits of respective locus tags in the GenBank accession numbers for SGI1 (AF261825), MGI*Vch*Hai6 (AXDR01000001), GI*Vch*Hai7 (ALDP01000008), GI*Vch*Bra1 (APFK01000082), and GI*Vch*Hai8 (ALED01000018). ACSSuT, resistance to ampicillin, chloramphenicol, spectinomycin/streptomycin, sulfamethoxazole, and tetracycline; Tm, trimethoprim resistance; Hg, mercury resistance.

Our group has previously described pVCR94, a type 2 IncA/C_2_ plasmid conferring MDR to the *V. cholerae* O1 El Tor strain responsible for the 1994 explosive cholera outbreak in Goma refugee camps (Democratic Republic of the Congo) (12). pVCR94 was used to characterize the main regulatory pathway that controls ACP conjugative transfer (12, 20, 21, 29). We have shown that *acaCD* encodes the master activator of ACP transfer that activates transcription of 18 ACP-borne genes and operons, including those coding for the conjugative machinery (21). In addition, AcaCD was shown to *trans*-activate excision and dissemination of MDR-conferring *Salmonella* genomic island 1 (SGI1) and unrelated MGI*Vmi*1 of *Vibrio mimicus* (Fig. 1) (20, 21, 29).

The presence of an MDR-conferring ACP in a 2012 *V. cholerae* isolate from Haiti has been reported only once to date (17). We report here the insidious role played by ACPs in the spread of MDR through mobilization of a new family of MDR-conferring genomic islands (GIs) in clinical non-O1/non-O139 *V. cholerae* isolates from Haiti. The presence of an ACP specifically triggers the excision and conjugative transfer of MGI*Vch*Hai6, the prototypical member of this new family of mobilizable GIs (MGIs). Further *in silico* analyses revealed the presence of MGI*Vch*Hai6-like elements in several environmental and clinical strains of *V. cholerae* isolated in North and South America and the Indian subcontinent. Our results demonstrate that ACPs have influenced the evolution of Haitian *V. cholerae* strains by propagating genomic islands, thereby allowing circulation of a vast reservoir of mobilizable MDR genes.

## RESULTS

### MDR determinants of *V. cholerae* HC-36A1 are neither carried on nor mobilized by SXT/R391 ICEs.

**FIG 2:**
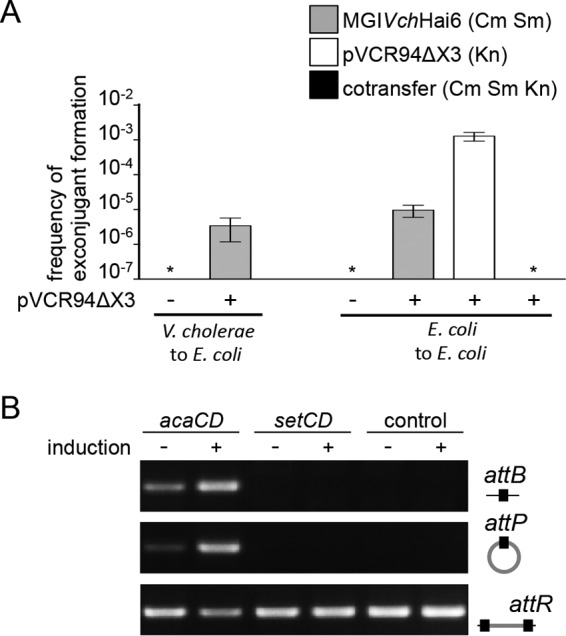
IncA/C-dependent excision and transfer of GIs. (A) MGI*Vch*Hai6 is mobilizable by ACPs. Inter- and intraspecific mobilization of MGI*Vch*Hai6 was assayed from *V. cholerae* to *E. coli* and from *E. coli* to *E. coli*, respectively. Interspecific transfer was done using *V. cholerae* HC-36A1 bearing pVCR94ΔX3 as the donor and *E. coli* CAG18439 as the recipient. Intraspecific transfer was performed using *E. coli* CAG18439 bearing pVCR94ΔX3 and MGI*Vch*Hai6 as the donor and *E. coli* MG1655 Rf as the recipient. Donor cells lacking pVCR94ΔX3 were used as a control. Exconjugants were selected for the acquisition of either MGI*Vch*Hai6, pVCR94ΔX3, or both. Transfer frequencies are expressed as the number of exconjugants per recipient CFU. Bars represent the mean and standard deviation values obtained from three independent experiments. Asterisks indicate the frequency of exconjugant formation below the detection limit (<10^−7^). Cm, chloramphenicol; Sm, streptomycin; Kn, kanamycin. (B) AcaCD specifically induces MGI*Vch*Hai6 excision. Excision was detected by PCR on genomic DNA to amplify chromosomal site *attB* and the *attP* site resulting from the excision of MGI*Vch*Hai6 in *E. coli*. Integrated MGI*Vch*Hai6 was detected by amplification of the *attR* site. Assays were done employing *E. coli* MG1655 Rf strains bearing the single-copy integrated IPTG-inducible p*acaDC*^3×FLAG^ vector (*acaCD*), the single-copy integrated IPTG-inducible p*setDC*^3×FLAG^ vector (*setCD*), or devoid of plasmid (control), without (−) or with (+) induction using IPTG.

*V. cholerae* HC-36A1 is a non-O1/non-O139 clinical isolate recovered in 2010 in the province of Port-au-Prince, Haiti at the beginning of the ongoing cholera outbreak in that country (4). Antibiotic susceptibility tests showed HC-36A1 is resistant to multiple antibiotics, including ampicillin, sulfamethoxazole, trimethoprim, chloramphenicol, kanamycin, streptomycin, and spectinomycin, and exhibits an inducible resistance to tetracycline when the strain is preexposed to subinhibitory concentrations of tetracycline (Table 1). The vast majority of Haitian outbreak strains carry ICE*Vch*Ind5 (also known as ICE*Vch*Hai1), a member of the SXT/R391 family of ICEs that mediates resistance to sulfamethoxazole, trimethoprim, chloramphenicol, and streptomycin (4, 30). Although most SXT/R391 ICEs are easily transferable between *V. cholerae* and *Escherichia coli*, our attempts to transfer resistance markers by conjugation, from *V. cholerae* HC-36A1 to either a rifampin-resistant derivative of *E. coli* MG1655 (MG1655 Rf) or the tetracycline-resistant *E. coli* strain CAG18439, did not generate any transconjugants (Fig. 2A). Analysis of the genome of HC-36A1 (GenBank accession no. AXDR01000001) confirmed the presence of an SXT/R391 ICE virtually identical to ICE*Vch*Hai2, an 83-kb ICE first identified in non-O1/non-O139 *V. cholerae* isolate HC-1A2 but lacking the typical antibiotic resistance gene cluster found in SXT/R391 ICEs (30). This result indicates that the resistance determinants carried by HC-36A1 are not carried by an SXT/R391 ICE or a genomic island that they can mobilize in *trans* or in *cis* (20, 31–33).

**TABLE 1:** MICs of 12 antibiotics against *V. cholerae* and *E. coli* with or without MGI*Vch*Hai6

Antibiotic	*V. cholerae* HC-36A1		*E. coli* CAG18439		*E. coli* CAG18439 MGI*Vch*Hai6	
	MIC (µg/ml)^a^	Phenotype^b^	MIC (µg/ml)^a^	Phenotype^b^	MIC (µg/ml)^a^	Phenotype^b^
Ampicillin	>120	R	<25	S	>800	R
Chloramphenicol	>32	R	10	S	>160	R
Ciprofloxacin	0.5	S	<0.13	S	<0.13	S
Erythromycin	<200	S	100	S	100	S
Gentamicin	2.5	S	<1.25	S	<1.25	S
Kanamycin	120	R	25	S	25	S
Nalidixic acid	<10	S	20	S	20	S
Rifampin	100	S	25	S	25	S
Streptomycin	>40	R	<50	S	>1,600	R
Spectinomycin	>80	R	100	S	>400	R
Sulfamethoxazole	ND	R	ND	S	ND	R
Tetracycline^c^	3–40	I	−	R	−	R
Trimethoprim	>40	R	<8	S	>256	R

a ND, not determined. Tests were done using solid agar plates.

b R, resistant; S, susceptible; I, inducible resistance.

c Tetracycline resistance of strain HC-36A1 ranges from 3 µg/ml to 40 µg/ml, without and with induction using 3 µg/ml of tetracycline as the inducible treatment, respectively. Tetracycline resistance tests were not carried out for the tetracycline-resistant strain CAG18439 (−).

### *V. cholerae* HC-36A1 holds a complex MDR-associated integron reminiscent of In104 from *Salmonella* genomic island 1.

Sequence analysis of the HC-36A1 genome aimed at localizing MDR determinants uncovered a new resistance gene cluster in chromosome I (Fig. 1). This locus contains a complex integron, In36A1, which exhibits structure and gene content relatively similar to those of the In104 resistance complex integron of SGI1 (Fig. 1) (34). Like In104, In36A1 contains the virtually identical resistance determinants *blaP* (β-lactams), *sul1* (sulfamethoxazole), *tetA*(G) (tetracycline), and *aadA2* (streptomycin/spectinomycin). In36A1 also contains a *floR* (florfenicol/chloramphenicol) variant sharing only 89% identity with *floR* of SGI1 and a trimethoprim resistance determinant sharing 93% identity with *dfrA23*, as a larger insertion between two *sul1* copies (Fig. 1).

In36A1 is surrounded by additional putative resistance loci: (i) a mercury resistance Tn*5053*-like transposon, Tn*6310*, and (ii) a gene cluster coding for an *hsd*-like type I restriction-modification (RM) system that can confer resistance to bacteriophage infections (35–37). Such accumulation of adaptive determinants within a 38-kb fragment hinted at the presence of a larger genomic island.

### In36A1 is part of a new family of genomic islands.

Analysis of the nearby sequence revealed proximity of a tRNA modification GTPase-encoding gene *trmE*, the 3′ end of which is the target site for SGI1 integration in *Salmonella enterica* (38–40). Located next to the *trmE* integration site is a gene, *int*, encoding a predicted tyrosine recombinase/integrase (VCHC36A1_0088) distantly related to the integrase of SGI1 (67% identity over 386 amino acid residues). Moreover, the gene adjacent to *int*, *xis*, codes for a predicted recombination directionality factor (RDF) (VCHC36A1_0087) sharing 37% identity over 106 amino acid residues with Xis of SGI1. Similar to SGI1, two imperfect direct repeats (differences underlined) were identified as part of the *attL* (TTCTGTATTGGGAAGTAA) and *attR* (TTCTGTATTGGCAAGTAA) attachment sites flanking the 47,437-bp genomic island MGI*Vch*Hai6. While MGI*Vch*Hai6 and SGI1 share distantly related recombination modules (*int*/*xis*) and closely related complex resistance integrons (In36A1 and In104, respectively), the remainder of the gene content is strikingly different (Fig. 1). Therefore, MGI*Vch*Hai6 is concluded not to be a SGI1-like element but rather a representative of a novel and large family of mobilizable genomic islands.

### ACPs trigger excision of MGI*Vch*Hai6 in *V. cholerae* HC-36A1.

Since we were unable to observe MDR transfer from ICE*Vch*Hai2-containing HC-36A1 to *E. coli*, we conclude that MGI*Vch*Hai6 is not mobilizable by SXT/R391 ICEs. On the other hand, the recent discovery of key regulators of ACP conjugation and the ability of the ACP-encoded positive regulator AcaCD to trigger excision of genomic islands SGI1 and MGI*Vmi*1 led us to consider ACPs are possible drivers of MGI*Vch*Hai6 mobility (20, 21, 29). *In silico* analysis of MGI*Vch*Hai6 revealed the presence of two divergent AcaCD binding motifs located in a 441-bp intergenic region. The first predicted AcaCD binding motif, GAAGTTCCCAAAAAGGGCAGTTCCAGCG (FIMO *P* value, 7.69 × 10^−10^), is located upstream of an operon-like cluster of three genes that likely code for a putative transcriptional regulator (VCHC36A1_0085), a DUF927 domain-containing protein of unknown function (VCHC36A1_0086), and Xis (Fig. 1). The second predicted AcaCD binding motif, CGTGTGCCCCAAAAGGGCACGAAGGCAG (FIMO *P* value, 3.26 × 10^−8^), is located upstream of a gene coding for a distant homolog of the mobilization protein MobI (27% identity over two fragments of 109 and 53 amino acid residues), a conserved key factor for conjugative transfer of ACPs (12, 20, 29). We also searched for binding motifs of the master activator of conjugation of SXT/R391 ICEs (SetCD) (41, 42), yet a SetCD binding motif with a FIMO *P* value below 10^−5^ could not be detected.

The presence of an AcaCD binding motif upstream of a putative *xis* gene strongly suggested that excision of MGI*Vch*Hai6 from the chromosome could be triggered by ACPs. To test this hypothesis, we introduced pVCR94ΔX3, a kanamycin-resistant derivative of ACP pVCR94, into *V. cholerae* HC-36A1 (21). To circumvent the presence of redundant kanamycin resistance in the two strains, as well as the possible retrotransfer of MGI*Vch*Hai6 from HC-36A1 to the donor cell, pVCR94ΔX3 was transferred to *V. cholerae* using as donor *E. coli* β2163, a *dapA* mutant auxotrophic for diaminopimelic acid (DAP) synthesis (43). The presence of pVCR94ΔX3, together with MGI*Vch*Hai6 in HC-36A1, was confirmed by growth on selective media and PCR amplification of specific DNA fragments—i.e., the promoter region of *traI* in pVCR94ΔX3 and a fragment in open reading frame 86 (ORF86) of MGI*Vch*Hai6. Employing PCR, we determined whether the presence of an ACP could trigger excision of MGI*Vch*Hai6, a mandatory step for transfer of a complete and functional genomic island. A fragment specific for an *attP* site resulting from excision of MGI*Vch*Hai6 from the chromosome was detected and sequenced, thereby confirming that excision of MGI*Vch*Hai6 occurred by recombination between the two imperfect direct repeats previously identified in the *attL* and *attR* attachment sites.

### MGI*Vch*Hai6 is *trans*-mobilizable by ACPs.

The excision of MGI*Vch*Hai6 in the presence of ACPs in the same cell prompted us to test whether pVCR94ΔX3 could mediate MGI*Vch*Hai6 conjugative transfer into *E. coli*. Therefore, *V. cholerae* HC-36A1 carrying pVCR94ΔX3 was used as a donor in mating assays using *E. coli* K-12 as a recipient*.* The presence of pVCR94ΔX3 specifically allowed the transfer of MGI*Vch*Hai6 and the associated MDR phenotype at a frequency of 3.5 × 10^−6^ exconjugant per recipient cell (Fig. 2A; Table 1). To exclude Hfr-like transfer of the MDR locus and subsequent integration by homologous or nonhomologous recombination into the chromosome of the recipient cell, the functionality of transferred MGI*Vch*Hai6 was verified in intraspecific transfer assays between *E. coli* strains (31, 44). While MGI*Vch*Hai6 was again unable to transfer autonomously, the presence of pVCR94ΔX3 triggered its transfer at a frequency of 1.0 × 10^−5^ exconjugant per recipient cell (Fig. 2A). In contrast, while pVCR94ΔX3 transferred at a rate of 1.3 × 10^−3^ exconjugant per recipient cell, the rate of cotransfer of both MGI*Vch*Hai6 and pVCR94ΔX3 was below the limit of detection, suggesting it might be a rare event (Fig. 2A).

### Master activator AcaCD specifically triggers excision of MGI*Vch*Hai6.

We further investigated whether excision of MGI*Vch*Hai6 was specifically dependent upon AcaCD. *E. coli* bearing MGI*Vch*Hai6 with or without a single chromosomally integrated copy of p*acaDC*^3×FLAG^, which expresses AcaCD under control of *P_tac_*, was used in PCR experiments to detect *attB* and *attP* sites resulting from MGI*Vch*Hai6 excision.

Excision assays revealed that the presence of AcaCD specifically triggers excision of MGI*Vch*Hai6, as shown by detection of specific *attB* and *attP* sites after IPTG (isopropyl-β-d-1-thiogalactopyranoside) induction (Fig. 2B). Excision of MGI*Vch*Hai6 was also observed without induction of *acaCD* expression, likely due to leaky transcription from *P_tac_* allowing sufficient production of AcaCD to induce the AcaCD-dependent promoters of MGI*Vch*Hai6 (21, 41). In contrast, *attB* and *attP* could not be detected in the strain expressing SetCD, the master activator of SXT/R391 ICE conjugation, thereby confirming that MGI*Vch*Hai6 excision is specifically triggered by ACP-encoded AcaCD (Fig. 2B). No spontaneous excision of MGI*Vch*Hai6 was detected using the control strain devoid of *acaCD-* or *setCD*-bearing plasmids.

### MGI*Vch*Hai6-like elements are widespread among *V. cholerae* epidemic and environmental strains.

**FIG 3:**
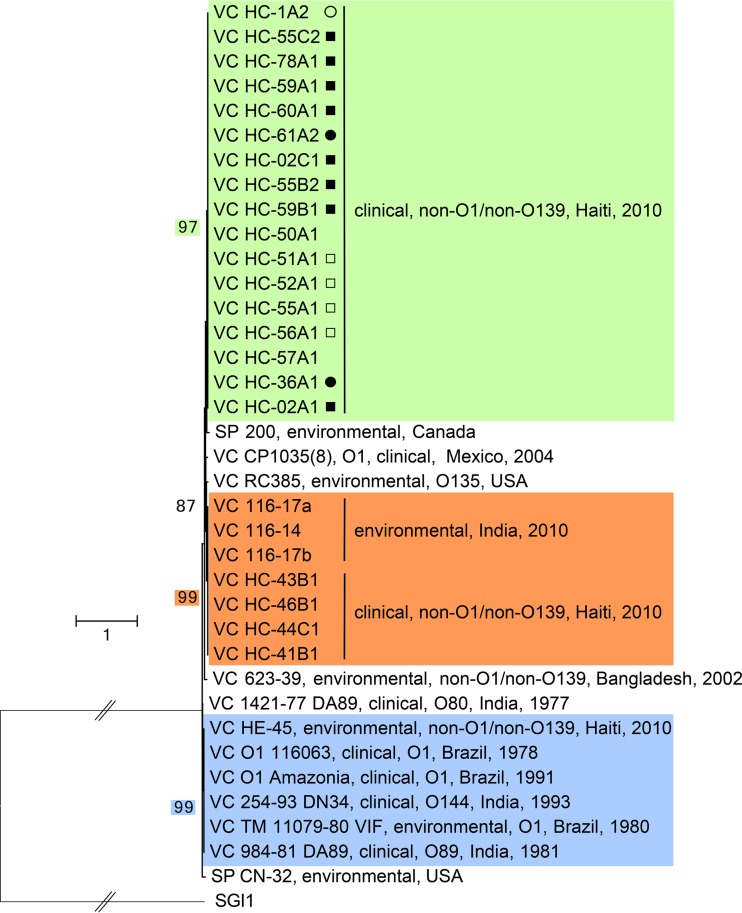
Molecular phylogenetic analysis of the *attL-int* locus of MGI*Vch*Hai6-related GIs by the maximum likelihood method. Based on the Hasegawa-Kishino-Yano model, the tree with the highest log likelihood (−5,618.1383) is shown (64). The percentage of trees in which the associated taxa clustered together is shown only for bootstrap values above 80. Subgroups were defined as clades supported by at least 95 bootstraps and are color coded as follows: strains containing MGI*Vch*Hai6-like elements are in green, GI*Vch*Hai7-like elements are in orange, and GI*Vch*Bra1 to GI*Vch*Hai8-like elements are in blue. Because analyses were done on nonassembled draft genome sequences, symbols indicate the confidence that the strain contains a complete element (solid circles, complete MGI*Vch*Hai6; open circles, partial detection of In36A1; solid squares and open squares, complete or partial detection, respectively, of Tn*6310* upstream of *attR*). The tree is drawn to scale, with branch lengths measured by the number of substitutions per site. For clarity, the length of the branch linking the tree to the outgroup (SGI1) was artificially divided by 4. *Vibrio cholerae* and *Shewanella putrefaciens* are abbreviated VC and SP, respectively.

To assess the diversity and abundance of MGI*Vch*Hai6-like elements among available genome sequences, *in silico* analysis was conducted using the *attL-int* DNA portion as a query to retrieve related genomic islands integrated into the 3′ end of *trmE*. Using this 1,352-nucleotide (nt) sequence in a search of the GenBank nucleotide collection database (nr/nt) targeting *Gammaproteobacteria* yielded only two significant matches, namely, against *Shewanella putrefaciens* genomes, one of which was previously detected in *S. putrefaciens* 200 as GI*Spu*1 (Fig. 3) (29). Further analyses targeting the whole-genome shotgun sequences and narrowing the analysis to *Vibrionaceae* revealed the presence of MGI*Vch*Hai6-related elements in the genome of various environmental and clinical *V. cholerae* strains (Fig. 3). Closer examination of these strains revealed that MGI*Vch*Hai6-related elements are not associated with a specific serotype or geographic location, because different strains were recovered from the Indian subcontinent, as well as from North and South America, including all non-O1/non-O139 clinical isolates from the 2010 outbreak in Haiti (Fig. 3). Interestingly, MGI*Vch*Hai6-related GIs were also detected in strains isolated from various locations in the 1980s, demonstrating that they had existed in *V. cholerae* populations prior to the 2010 Haitian cholera outbreak.

The evolutionary history of the *attL*-*int* locus of MGI*Vch*Hai6-related GIs was inferred using sequences recovered from BLAST analyses and using SGI1 as an outgroup (Fig. 1 and 3). Three strongly supported clades of elements were delineated. One representative GI of each cluster was chosen for schematic representation: MGI*Vch*Hai6 from strain HC-36A1 (green cluster), the 19-kb element GI*Vch*Hai7 from strain HC-43B1 (orange cluster), and GI*Vch*Bra1 and GI*Vch*Hai8 (17.6 kb) found in their respective strains 116063 and HE-45 (blue cluster) (Fig. 1 and 3).

All *V. cholerae* isolates possessing MGI*Vch*Hai6-like elements were clinical non-O1/non-O139 strains from the 2010 Haitian outbreak (Fig. 3, green cluster). Because analyses had been done on nonassembled draft genome sequences, we were not able to determine the exact structure of each member, but detection ranged from the complete MGI*Vch*Hai6 to partial detection of Tn*6310* upstream of *attR*.

While they are very different in size, GI*Vch*Hai7 shares similar structure with the green group prototype, MGI*Vch*Hai6 (Fig. 1). In particular, the two GIs carry closely related recombination modules (*attL* to *int*/*xis*), as well as nearly identical right ends (*pinE* to *attR*) (Fig. 1). Nevertheless, these two elements are concluded to be distantly related as they share limited homology in the region containing the two AcaCD-dependent promoters and only in the 3′ end of the *mobI* gene. Surprisingly, in spite of the fact that GI*Vch*Hai7 and MGI*Vch*Hai6 carry a gene predicted to encode a DUF927 protein upstream of *xis*, their respective ORFs share no significant nucleotide similarity (Fig. 1). GI*Vch*Hai7 encodes a putative *hsd* type I RM system but lacks the resistance gene cluster insertion in *pinE* in MGI*Vch*Hai6, suggesting the entire MDR-conferring gene cluster of MGI*Vch*Hai6 and SGI1 is mobile and highly plastic.

The two representative members of the third group, GI*Vch*Bra1 and GI*Vch*Hai8, share identical nucleotide sequence, with the exception of three sites of single nucleotide polymorphisms (SNPs) in *int* and one SNP in the gene coding for the DUF927 protein (Fig. 1). These elements are closely related to GI*Vch*Hai7 as they share almost identical left and right ends. While the regions between *mobI* and *pinE* code for a type I *hsd* RM system in both GI*Vch*Bra1 and GI*Vch*Hai8 and GI*Vch*Hai7, their organizations differ significantly.

## DISCUSSION

The availability of a massive amount of sequencing data for bacterial genomes, combined with functional data on various mobile genetic elements that has accumulated over the last four decades, allowed an extensive epidemiological survey of MDR genetic determinants circulating among pathogenic and environmental bacteria. Previous studies pointed out the major role of mobile genetic elements in propagation of MDR, notably conjugative elements such as conjugative plasmids and ICEs (5, 45–47).

Recent progress in deciphering the biology of SXT/R391 ICEs and ACPs has refined our understanding of the biology of these major drivers of the distribution of MDR among the *Gammaproteobacteria* (7, 11, 21, 41, 48–50). In particular, both SXT/R391 ICEs and ACPs were shown to *trans*-mobilize many MGIs conferring adaptive traits to their bacterial host, including MDR (21, 29, 31, 32, 51). These MGIs were found to be activated by the master activator of SXT/R391 ICEs (SetCD) or ACPs (AcaCD), thus being part of the extended regulatory network of these autonomous conjugative elements (20). Functional studies of the biology of SXT/R391 ICE-dependent MGIs has revealed key features, but the precise mechanisms allowing dissemination of ACPs-dependent MGIs remain largely unknown (31, 32). Based on recent findings, it is hypothesized that the superfamily of MGIs bearing AcaCD-activated homologs of *mobI* and *xis*, including the MGIs described here, as well as other elements previously discovered (MGI*Vmi*1, GI*Vmi*2, GI*Vpa*1, and GI*Spu*1), share a mechanism of mobilization (21, 29). Transcriptional data obtained on MGI*Vmi*1 showed that AcaCD specifically triggers transcription of a recombination directionality factor (RDF)-encoding gene (*xis*), allowing excision of the MGI (21). The presence of an AcaCD-activated *mobI* homolog strongly suggests that, as shown for SXT/R391 ICEs and ACPs, the upstream intergenic region likely constitutes the origin of transfer (*oriT*) of the MGI (12, 20, 52). The current working model surmises that, upon AcaCD activation, MobI of the MGI likely recognizes its cognate *oriT* and recruits the ACP MobI-less relaxosome that processes the DNA to transfer one strand of the MGI from the donor cell to the recipient (20). Once in the recipient cell, the complementary strand is synthesized, and constitutive expression of the *int* gene allows site-specific integration of the MGI, regardless of the presence of helper ACP. Functional characterization of each gene and precise determination of the *oriT* locus are ongoing using different representative of the above-mentioned MGIs.

Current sequence analyses indicate that the size of the core sequence of the MGI family is ca. 8 kb and that it corresponds to DNA regions from *attL* to *mobI* and from *pinE* to *attR* (Fig. 1). *pinE*, which codes for a putative recombinase/invertase, is unlikely to be involved in excision, integration, or mobilization of the MGIs as it serves as an insertion site for the In36A1 resistance gene cluster, which moves by transposition (34, 53). While MGI*Vch*Hai6 carries antibiotic resistance genes, as well as a mercury resistance transposon, the majority of adaptive traits encoded by related GIs appear to be limited to RM systems (Fig. 1) (20). The presence of RM systems is considered to be ubiquitous in such GIs, likely conferring strong selective advantage to their host against bacteriophages, which thrive in aquatic environments. Nevertheless, a single event of transposition could lead to acquisition of an In104-like element and its associated MDR phenotype. Moreover, the presence of an integron likely allows further antibiotic resistance gene acquisition from, and exchange with, other integrons carried by other mobile genetic elements (46).

Although MGIs have been recently discovered, these elements are not specific to recent multidrug-resistant isolates of bacteria. For instance, GI*Vch*Bra1 was found in the genome of a *V. cholerae* strain isolated in 1978 in Brazil and appears to have circulated since then, with isolation of its sibling GI*Vch*Hai8 in 2010 in Haiti (Fig. 1 and 3). Such sequence conservation implies a very recent transfer event or a highly active element that does not accumulate mutations while quiescent in the chromosome of its bacterial host (54). Further large-scale analyses of sequence databases facilitated by very rapid addition of many additional bacterial genomes provided by massive sequencing, together with ongoing functional dissection of their biology, will most likely clarify the dynamics of these MGIs.

SXT/R391 ICEs were defined as major drivers of MDR among *V. cholerae* strains, generating a strong bias toward their detection in epidemiological reports (9, 10, 55). More recent studies highlight the presence of ACPs as MDR determinants in clinical and environmental *V. cholerae* strains (12–18). While ACPs are widely distributed in many species of pathogenic bacteria, their occurrence in *V. cholerae* is less well characterized. It is plausible that ACPs are not stably maintained in *V. cholerae* or that the role of ACPs in MDR acquisition by *V. cholerae* was overlooked. In agreement with the first hypothesis, sequence analyses of *V. cholerae* strains analyzed in this study using the IncA/C_2_ *repA* gene revealed that only the Indian environmental strains 116-14, 116-17a, and 116-17b contain an ACP (18). Nevertheless, ACPs likely have a more insidious role in the dissemination of MDR by not remaining in *Vibrio* strains but efficiently promoting circulation of MDR-associated MGIs and the adaptive traits that they may confer. In the future, circulation of MGI*Vch*Hai6 and related elements driven by ACPs should be monitored as they could enhance and accelerate dissemination of MDR in *V. cholerae* and related pathogens.

## MATERIALS AND METHODS

### Bacterial strains and media.

The bacterial strains and plasmids used in this study are described in Table 2. The strains were routinely grown in lysogeny broth (LB-Miller; EMD) at 37°C in an orbital shaker/incubator and preserved at −80°C in LB broth containing 15% (vol/vol) glycerol. For *E. coli*, antibiotics were used at the following concentrations: ampicillin, 100 µg/ml; chloramphenicol, 20 µg/ml; erythromycin, 200 µg/ml; gentamicin, 10 µg/ml; kanamycin, 50 µg/ml; nalidixic acid, 40 µg/ml; rifampin, 50 µg/ml; spectinomycin, 50 µg/ml; streptomycin, 200 µg/ml; sulfamethoxazole, 160 µg/ml; tetracycline, 12 µg/ml; and trimethoprim, 32 µg/ml. For *V. cholerae*, antibiotics were used at the following concentrations: chloramphenicol, 2 µg/ml; kanamycin, 30 µg/ml; streptomycin, 10 µg/ml; and tetracycline, 10 µg/ml. When required, bacterial cultures were supplemented with 0.3 mM dl-2,6-diaminopimelic acid (DAP) or 0.02 mM isopropyl-β-d-1-thiogalactopyranoside (IPTG). Antibiotic susceptibility profiling and MIC determination were performed using broth dilution tests (56).

**TABLE 2:** Strains and plasmids used in this study

Strain or plasmid	Relevant genotype or phenotype^a^	Reference(s)
Strains		
*V. cholerae* HC-36A1	Clinical, non-O1/non-O139, Haiti (Tabarre) 2010 (Ap Cm Kn Sp Sm Su Tc Tm)	4
* E. coli*		
MG1655 Rf	Rf^r^ derivative of MG1655 (Rf)	52
CAG18439	MG1655 *lacZU118 lacI42*::Tn*10* (Tc)	63
β2163	(F^−^) RP4-2-Tc::Mu *dapA*::(*erm*-*pir*) (Kn Em)	43
Plasmids		
pVCR94ΔX3	Kn^r^ derivative of IncA/C plasmid pVCR94 (Kn Su)	21
p*acaDC*^3×FLAG^	pAH56::*acaDC*^3×FLAG^ (Kn)	21
p*setDC*^3×FLAG^	pAH56::*setDC*^3×FLAG^ (Kn)	21, 41

a Ap, ampicillin; Cm, chloramphenicol; Em, erythromycin; Kn, kanamycin; Rf, rifampin; Sm, streptomycin; Sp, spectinomycin; Su, sulfamethoxazole; Tc, tetracycline; Tm, trimethoprim.

### Bacterial conjugation assays.

Conjugation assays were performed as described by Carraro et al. (12). Donors, recipients, and exconjugants were selected on LB agar plates containing the appropriate antibiotics. If needed, additional controls were done on isolated clones using selective media, including thiosulfate-citrate-bile salts-sucrose (TCBS) agar (Difco) for identification of *V. cholerae* and PCR amplification of the promoting region of pVCR94ΔX3 and/or an internal fragment of ORF86 of MGI*Vch*Hai6 using primer pairs promvcrx060traIpstI.for/promvcrx060traIpstI.rev and GIVch1verif.for/GIVch1verif.rev, respectively (Table 3).

**TABLE 3:** Primers used in this study

Name	Nucleotide sequence (5′ to 3′)	Reference
thdF_attBF	TATAGCCCAGCAACACCTTA	This study
int1_attPR	GGATCTCGTTTATGTATGCTGA	This study
43_attPF	GCAATTAATGATAAAGACGGGTA	This study
int2_attBR	GGTATAACCGTGGTCATAAATGA	This study
EcU7-L12.for	ACATCTACAACAGGGCAAAG	38
Ec104D.rev	AACCATTTTGAGGTCACACA	38
promvcrx060traIpstI.for	NNNNNNCTGCAGCATCAAAAATTGTCGATGA	21
promvcrx060traIpstI.rev	NNNNNNCTGCAGCTATCGTATTTCTCGTCGCTA	21
GIVch1verif.for	TGCCATGGTCCGAAGAAGAGTC	This study
GIVch1verif.rev	AATCCGCGTTTTATAGGTTCCC	This study

### Molecular biology methods.

All enzymes used in this study were purchased from New England Biolabs. PCR assays were performed employing the primers listed in Table 3 under the following conditions: (i) 3 min at 94°C; (ii) 30 cycles of 30 s at 94°C, 30 s at the appropriate annealing temperature, and 1 min/kb at 68°C; and (iii) 5 min at 68°C. When necessary, PCR products were purified using an EZ-10 spin column PCR product purification kit (Biobasic) according to the manufacturer’s instructions. Sequencing reactions were performed by the Plateforme de Séquençage et de Génotypage du Centre de Recherche du CHUL (Québec, QC, Canada).

### Detection of MGI*Vch*Hai6 excision.

Excision of MGI*Vch*Hai6 was detected by PCR on genomic DNA of the appropriate strains, using the primers listed in Table 3. The *attR* site was amplified using primer pair 43_attPF/int2_attBR in *V. cholerae* and pair 43_attPF/Ec104D.rev in *E. coli*. The *attB* chromosomal site was detected using thdF_attBF/int2_attBR in *V. cholerae* and EcU7-L12.for/Ec104D.rev in *E. coli* (38). The *attP* site carried by the extrachromosomal circular form of MGI*Vch*Hai6 was amplified using the primer pair 43_attPF/int1_attPR.

### Sequence annotations.

Detection of the *attL* and *attR* attachment sites flanking MGI*Vch*Hai6 and related genomic islands was carried out using the software YASS to identify the direct repeats (57). Genes were predicted using the RAST pipeline (58), and spurious annotations were manually curated. Antibiotic resistance determinants were detected using ResFinder 2.1 (https://cge.cbs.dtu.dk/services/ResFinder/) and The Comprehensive Antibiotic Resistance Database (http://arpcard.mcmaster.ca/?q=CARD/tools/RGI). Precise locations of AcaCD binding motifs were searched using FIMO (59) against the sequence of MGI*Vch*Hai6, GI*Vch*Hai7, and GI*Vch*Bra1 with the AcaCD and SetCD MEME logos, as described elsewhere (21, 41).

### Phylogenetic analyses.

Molecular phylogenetic analysis of the *attL-int* locus was performed in MEGA6 (60). The 1,351-bp nucleotide sequence of MGI*Vch*Hai6 encompassing 18 nt at the end of *trmE* that correspond to the *attL* site direct repeat and ending at the top codon of *int* was used to search for homologous sequences in the GenBank nucleotide collection database (nr/nt targeting *Gammaproteobacteria* and WGS targeting *Vibrionaceae*) using nucleotide BLAST (61). The corresponding sequence in *Salmonella* genomic island 1 (SGI1) was manually added to the data set. Phylogenetic analyses were computed using a nucleotide alignment generated by MUSCLE (62). The evolutionary history was inferred by using the maximum likelihood method. Initial trees for the heuristic search were obtained automatically by applying the Neighbor-Join and BioNJ algorithms to a matrix of pairwise distances estimated using the maximum composite likelihood (MCL) approach and selecting the topology with superior log likelihood value. A discrete gamma distribution was used to model evolutionary rate differences among sites (5 categories [+G, parameter = 0.7615]). The analysis involved 37 nucleotide sequences. All positions with less than 95% site coverage were eliminated, providing a total of 1,350 positions in the final data set.
